# Structural Characterization of Degraded *Lycium barbarum* L. Leaves’ Polysaccharide Using Ascorbic Acid and Hydrogen Peroxide

**DOI:** 10.3390/polym14071404

**Published:** 2022-03-30

**Authors:** Majida Al-Wraikat, Yun Liu, Limei Wu, Zeshan Ali, Jianke Li

**Affiliations:** College of Food Engineering and Nutritional Science, Shaanxi Normal University, Xi’an 710119, China; majida@snnu.edu.cn (M.A.-W.); yunyun800924@snnu.edu.cn (Y.L.); iwulimei@163.com (L.W.); zeshan@baraniinstuite.edu.pk (Z.A.)

**Keywords:** *Lycium barbarum* L. leaves, polysaccharide, degradation, ascorbic acid, microstructure

## Abstract

Plant-derived polysaccharide’s conformation and chain structure play a key role in their various biological activities. *Lycium barbarum* L. leaves’ polysaccharide is well renowned for its health functions. However, its functional bioactivities are greatly hindered by its compact globular structure and high molecular weight. To overcome such issue and to improve the functional bioactivities of the polysaccharides, degradation is usually used to modify the polysaccharides conformation. In this study, the ethanol extract containing crude *Lycium barbarum* L. leaves’ polysaccharide was first extracted, further characterized, and subsequently chemically modified with vitamin C (Ascorbic acid) and hydrogen peroxide (H_2_O_2_) to produce degraded *Lycium barbarum* L. leaves’ polysaccharide. To explore the degradation effect, both polysaccharides were further characterized using inductively coupled plasma mass spectrometry (ICP-MS), gas chromatography–mass spectrometry (GC–MS), Fourier transform infrared spectroscopy (FTIR), nuclear magnetic resonance (NMR), high performance gel permeation chromatography (HPGPC), and scanning electron microscope (SEM). Results shown that both polysaccharides were rich in sugar and degradation had no significant major functional group transformation effect on the degraded product composition. However, the molecular weight (Mw) had decreased significantly from 223.5 kDa to 64.3 kDa after degradation, indicating significant changes in the polysaccharides molecular structure caused by degradation.

## 1. Introduction

The leaves of *Lycium barbarum* L. (LL), also known as Goji, which is a plant belonging to the family *Solanaceae*, are regarded in traditional Chinese medicine as a medical herb for eternal youth and long life, a nourishing ingredient, and a tonic to reduce the risk of arteriosclerosis and essential arterial hypertension [1,2]. Recently, LL have attracted significant attention due to the very high content of bioactive substances and nutrients. The major functional components, including flavonoids, carotenoids, and polysaccharides, have been reported to be closely associated with health-enhancing effects and antioxidant activities [3,4,5,6].

*Lycium barbarum* L. polysaccharides (LP) is one of the major ingredients responsible for those biological activities in LL. Previous studies have demonstrated that LP has several various important biological activities. LP had potent free radical-scavenging properties and showed considerable inhibitory activities for antioxidant, immunomodulation, antitumor, neuroprotection, radioprotection, anti-diabetes, hepatoprotection, anti-osteoporosis, and antifatigue agents [7,8,9,10,11,12]. Furthermore, LP has been proven to be a rich nutritional supplement and a food additive [13,14], and showed great potential in green synthesized nanoparticles [15,16,17]. Nevertheless, LP bioactivity is considerably low, and greatly affected by its structural confirmation. This is due to its high molecular weight (Mw) and compact globular structure, which has wrapped abundant endogenous minerals inside itself by ionic interactions with uronic acid, which is not conducive to the dissociation and release of mineral elements [18,19].

Several methods are used to enhance polysaccharide’s functional bioactivities, for example: enzymatic hydrolysis, sulphated modification, and carboxymethylated modification [20]. However, degradation using hydrogen peroxide (H_2_O_2_) and Vitamin C (Vc) is preferred due to its high efficiency, moderate reaction conditions, and negligible damage to sugar structures [19,21]. The degradation chemical reaction system breaks the glycosidic links and transforms the polysaccharides confirmation, freeing and allowing more elements to be dissociated and released [18,22,23]. Nevertheless, this degradation method has rarely been fully exploited and the reaction system influence of degradation on polysaccharides conformation, structure, and characteristics has not been fully investigated.

For this purpose, the ethanol extract containing LP was first separated, deproteinized, and purified to produce the crude LP (CLP). Subsequently, CLP were chemically modified with Vc (Ascorbic acid) and H_2_O_2_ to produce the degraded LP (DLP). To explore the degradation effect, both CLP and DLP were further characterized using inductively coupled plasma mass spectrometry (ICP-MS), gas chromatography–mass spectrometry (GC–MS), Fourier transform infrared spectroscopy (FTIR), nuclear magnetic resonance (NMR), high performance gel permeation chromatography (HPGPC), and scanning electron microscope (SEM). Finally, results of CLP and DLP were plotted, tabulated, and compared. The action mechanism of degradation was also elucidated by analysing the influence on the structural conformation of LP.

## 2. Materials and Methods

### 2.1. Materials

*Lycium barbarum* L. leaves were harvested in Ningxia, China in August 2020. The leaves were carefully washed, dried at 60 °C for 24 h, grinded into a fine powder (40 mesh), and stored at room temperature until use. The mineral standards were obtained from the National Institute of Metrology, China. Monosaccharide standards and uronic acid standards were purchased from Sigma (St. Louis, MO, USA). Trifluoroacetic acid (TFA) was obtained from Merck (Darmstadt, Germany). Triethylamine was from Xi’an Reagent Plant (Xi’an, China). Vitamin C (Vc), H_2_O_2_, and other materials and regents were of analytical grade and the highest grade available.

### 2.2. LP Extraction

LP was prepared following the method described by Liu et al. [7] with minor modifications. Briefly, LL powder (50 g) was dissolved in distilled water (1000 mL) and incubated for 1 h at 120 °C, filtered, and the filtrate was concentrated using a rotary evaporator at 70 °C. The concentrated solution was precipitated overnight using 4 volumes of ethanol (99%) and further repeatedly centrifuged and lyophilized.

### 2.3. Protien Removal

To reduce the complexity of the extract and exclude the effect of proteins in subsequent experiments, proteins in the LP extract were removed based on the following procedures: LP were dissolved in distilled water to a final concentration of 5%, to which 1/4 volume of Sevage reagent (chloroform: n-butanol = 4:1, *v*/*v*) was added, followed by 30 min shaking. The shake was then mixed with sodium hydroxide powder (2 g) dissolved in 100 mL distil water, and the pH was adjusted to (9–10) using a test paper. Protease was then added and the mix was incubated in a hot bath at 55 °C for 4 h. The mixture was then centrifuged at 6000× *g* for 8 min and the bottom layer of denatured proteins was discarded. The supernatant was then removed, filtered using 0.8 and 0.45, dried and dialyzed for 3 days, 6 h per day. Finally, 4 volumes of ethanol were added and incubated overnight. After centrifugation, the precipitates were obtained, and repeatedly frozen and lyophilized. LP protein contents were checked using Lowry’s method [24].

### 2.4. LP Purification

LP was purified by using the DEAE-cellulose-52 column as described by Guo et al. [25], with some modifications. Procedures were as follows: First, 1 g DEAE-cellulose-52 was weighed and placed in a 25 mL cylinder. A total of 10 mL deionized water was added to the cylinder to soak for 24 h. The swelling volume was calculated. Appropriate amount of DEAE-cellulose-52 was weighed based on the swelling volume and added to a beaker containing deionized water to soak for 24 h. Water was replaced twice during soaking and suspended matter was removed and discarded. Second, excess water was removed from the soaked and expanded DEAE-cellulose-52 with a suction filter and 0.5 mol/L NaOH solution was added, mixed well, and soaked for 1 hr. The cellulose was washed with deionized water until neutral pH was reached. Then 0.5 mol/L HCl solution was added and soaked for 1 h, followed by washing with deionized water till neutral. Then 0.5 mol/L NaOH solution was added, incubated for 1 h, and washed with deionized water to neutral, followed by ultrasonic degassing. Third, a chromatography column (2.3 × 60 cm^2^) was fixed with filter paper placed at the bottom. A total of 1/3 volume of deionized water was added to the column and the pre-treated DEAE-cellulose-52 was added slowly to the column (preventing the production of bubbles). After sedimentation, the remaining cellulose was added to the column to prevent the filler from delaminating. When DEAE-Cellulose-52 was sedimented to a height of 3–5 cm from the top of the column, loading process was finished. Deionized water was added to the column and equilibrated over-night. Fourth, 0.1 g deproteinized LP extract was added to 5 mL deionized water and fully dissolved, and the extract solution was loaded slowly onto the equilibrated chromatography column. Tube was connected to the lower part of the column and automatic collector was setup with a flow rate of 1 mL/min. The column was sequentially eluted with deionized water, 0.05, 0.1, 0.2, and 0.4 M NaCl solution, and elutes were collected.

The content of sugar in each tube of the eluate was determined by the phenol-sulfuric acid method [26]. Results showed that the highest content of polysaccharide was found in the 0.1 M NaCl eluate and all eluates within the range of 0.1 M NaCl elution were pooled together for further study. The pooled eluates were lyophilized and the product is named hereinafter CLP.

### 2.5. Degradation of LP

DLP was prepared using the degradation method described by Zhang et al. [27]. Briefly, 1 g of CLP solution in 100 mL distilled water was heated up to 30 °C. Subsequently, H_2_O_2_ (0.5 mL) and Vc (0.88 g) were added at a molar ratio of 1:1. After magnetic stirring at room temperature for 2 h, the reactant was dialyzed, precipitated with alcohol (80%), and repeatedly lyophilized. The resultant was then obtained and labelled, and is named hereinafter DLP. The degradation degree was determined as follows:

$$Degradation\;Degree\;\left(\%\right)=\frac{Mw_{0}-Mw_{c}}{Mw_{0}}\times 100$$

where $Mw_{0}$ is the initial Mw of the polysaccharides (CLP); and $Mw_{c}$ is the Mw of the polysaccharides at given H_2_O_2_ and Vc concentrations.

### 2.6. Uronic Acid Content

The content of uronic acid was determined according to the method described by Ren et al. [18]. A total of 0.1 mg/mL galactonic acid solution was accurately prepared with reference standard and each LP sample solution was prepared at 1 mg/mL. A total of 75 mg carbazole was dissolved in 50 mL anhydrous ethanol to prepare a 0.15% carbazole solution. A total of 0.478 g sodium tetraborate was accurately weighed and dissolved in 100 mL concentrated sulfuric acid. A total of 1 mL sample solution was added to a test tube with a cap, and 5 mL sodium tetraborate-sulphuric acid solution was added to the test tube on ice. The mixture in the test tube was heated in boiling water for 20 min. The tube was removed and immediately cooled in ice water, and 0.2 mL of 0.15% carbazole solution was added. The tube was shaken well and placed at room temperature for 2 h. Absorbance at 523 nm was determined.

### 2.7. Mineral Elements Content

A total of 1 g of each CLP and DLP samples were accurately weighed and added in a 100 mL dried flask. A total of 10 mL concentrated nitric acid was added to the flask and placed overnight. The next day, the mixture was heated on an electric furnace to dissolve the sample until the solid disappeared. Then, 10 mL concentrated nitric acid and 5 mL perchloric acid for further digestion. The mixture was heated till the solution became transparent with white smoke, and then transferred to a 50 mL volumetric flask after cooling. The volume was fixed with 0.5% nitric acid to the scale [28]. The contents of calcium, iron, zinc, and magnesium were then determined using inductively coupled plasma mass spectrometry (ICP-MS) [29]. The determination of ash and moisture contents were performed following the methods no. 930.05 and 930.04 described by the Association of Analytical Chemists (AOAC), respectively [30].

### 2.8. Monosaccharide Analysis

CLP and DLP monosaccharide compositions were measured by gas chromatography (GC-2010, Shimadzu Corporation, Kyoto, Japan). Briefly, 10 mg were dissolved in 2 mL trifluoroacetic acid (TFA) and hydrolysed at 120 °C for 2 h. After drying using a rotary evaporator at 70 °C, TFA was removed by repeatedly adding methanol (2 mL). Finally, monosaccharaides were analysed according to the method described in [7] using gas chromatograph equipped with a Rtx-5 column and flame ionization detector (FID). The monosaccharaides molar ratios were determined based on their peak areas and standards [31].

### 2.9. Fourier Transform Infrared Spectrometer

Fourier transform infrared spectrometer (FTIR; Bruker Vertex 70, Bruker Optics, Ettlingen, Germany) was used for scanning. Briefly, samples were loaded after the instrument was preheated and stabilized, and the scanning was performed at a range of 4000–400 cm^−1^ with a resolution of 4 cm^−1^ and scanning times of 70.

### 2.10. Nuclear Magnetic Resonance Spectra

^1^H and ^13^C NMR spectra of both CLP and DLP were obtained with an NMR spectrometer (JNM-ECZ400R/S1, JEOL Ltd., Tokyo, Japan) using a sample of 20 mg/mL in D_2_O (0.5 mL, 99.9%). Chemical shifts are reported in ppm.

### 2.11. Molecular Weight (Mw) and LP Conformation

CLP and DLP Mw and structure conformation were determined by high performance gel permeation chromatography (HPGPC; VISCOTEK TM, Malvern, UK) equipped with both a refractive index detector (RID) and a multi-angle laser scattering detector (MALSD). For each LP, 1 mg was dissolved in 1 mL deionized water, and then filtered using 0.45 μm and 0.22 μm syringe filters for aqueous solutions. A total of 20 μL of the sample was loaded and NaNO_3_ was used as the mobile phase. The flow rate was set to 0.5 mL/min at a temperature of 35 °C. A calibration curve was produced using Dextran T-series standards of different molecular weights. The Mw of both LPs were estimated with reference to the calibration curve using the RID (35 °C). Simultaneously, the gyration radius of LP molecules was determined using HPGPC and MALSD (35 °C).

### 2.12. Scanning Electron Microscopy (SEM) Analysis

Surface analysis of CLP and DLP were featured using scanning electron microscopy (SEM; SU8010; Hitachi, Tokyo, Japan). Images of both LPs were prepared as dry and wet status. The absolutely dried LP samples was sputtered in advance for the SEM observation with the magnification set at 1000× *g*.

### 2.13. Statistical Analysis

Data were analysed using SPSS 11.0 (SPSS Inc., Chicago, IL, USA) software, and were presented as mean ± SD at *p* < 0.05. Analysis of variance was used to evaluate mean values differences. All determinations were performed in at least triplicate and results were expressed in dry weight (dw).

## 3. Results

### 3.1. LP Characteristics

**Figure 1:**
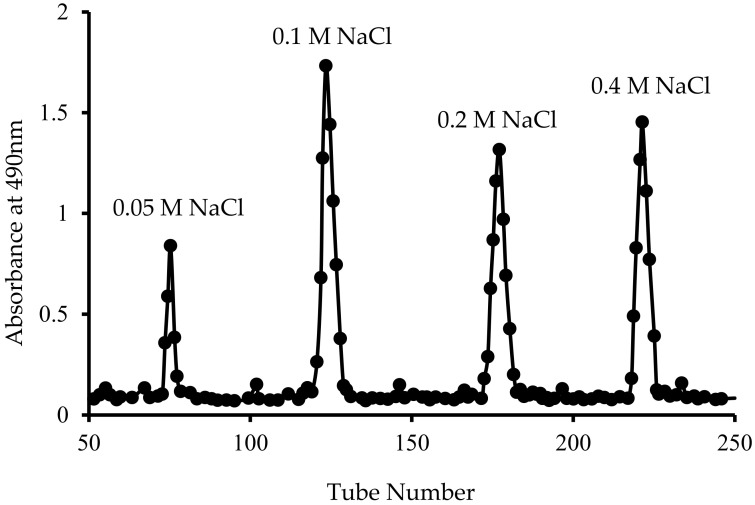
Elution curve of polysaccharide fractions obtained using a DEAE-C 52 column eluted with water and NaCl solution.

A total of four major fractions of LP was obtained after purification using DEAE-cellulose-52 column isolation, as shown in Figure 1, in which the fraction eluted with 0.1 M NaCl showed the highest polysaccharide content (94.0 ± 6.8%), and therefore was used as CLP. It showed a single, symmetric peak after further treatment using Sephadex G-100. Previous study on the 0.1 M NaCl elution found 76.6% polysaccharide [18] while a higher content of 96% was reported by Zhang et al. [22] for the 0.4 M NaCl elute, which has good consistency with the current presented study.

### 3.2. LP Proximate Composition

Comparison analysis on the proximate composition of CLP and DLP determined by this study are shown in Table 1 below. The protein content of CLP was (2.5 ± 0.1%), indicating highly purified polysaccharide. In addition, CLP contained high levels of total sugar (94.0 ± 6.8%) and uronic acid (13.7 ± 1.7%). Ash and moisture contents were of (14.7 ± 1.2%) and (3.9 ± 0.2%), respectively. These results were consistent with previously reported values on LP, except for uronic acid, which is much lower than the 40.6% reported by [18]. Mineral content of CLP presented in Table 1 showed high levels of calcium (8.8 ± 1.1 mg/g), iron (2.29 ± 0.32 mg/g), zinc (1.51 ± 0.06 mg/g), and magnesium (3.96 ± 0.22 mg/g). These results differ significantly from those previously reported by [18] for calcium (104.7 mg/g) and zinc (0.3 mg/g). However, calcium value was consistent with that of (8.6 mg/g) reported by [22].

On the other hand, DLP proximate compositions and mineral content (shown in Table 1) were slightly different to those obtained in CLP. The protein content of DLP (2.67 ± 0.1%) indicated the same highly purified degree. Similarly, DLP contained high levels of total sugar (89.2 ± 7.9%) and uronic acid (15.2 ± 0.7%). Mineral content of DLP also showed high levels of calcium (7.5 ± 0.2 mg/g), iron (1.96 ± 0.17 mg/g), zinc (1.42 ± 0.3 mg/g), and magnesium (3.63 ± 0.4 mg/g).

### 3.3. Monosaccharaides Composition

**Figure 2:**
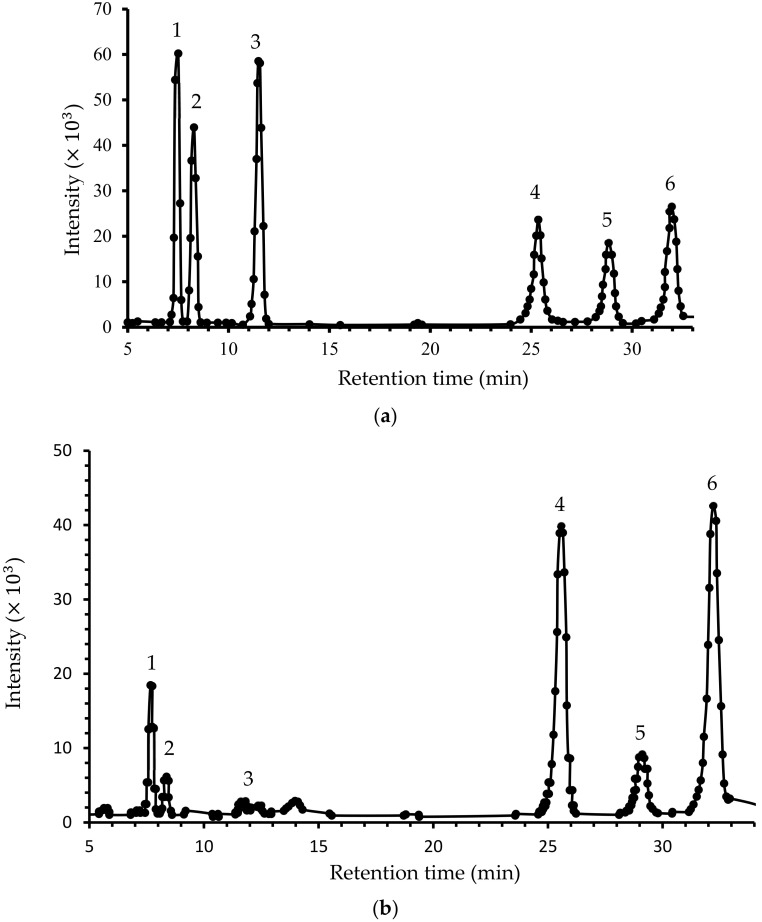
Monosaccharide analysis of (**a**) CLP and (**b**) DLP, where; 1, 2, 3, 4, 5, and 6 are mannose, ribose, glucuronic acid, glucose, xylose, and galactose, respectively.

Gas chromatograph spectroscopic analysis, presented in Figure 2, showed that CLP is composed of six monosaccharaides, namely, mannose, ribose, glucuronic acid, glucose, xylose, and galactose, at ratios of 4.1, 1.7, 3.1, 1.5, 3.26, and 1.9, respectively. These results agree with our previous study [14], while they differ from that of LP eluted with 0.4 M NaCl and 0.2 NaCl, where arabinose and rhamnose were detected instead of mannose and glucuronic acid [22]. In general, the carbohydrate composition of CLP is similar to that of pectin polysaccharides. However, in CLP, higher ribose content was detected [32].

Degradation effect on monosaccharaides composition is shown in Figure 2b. The analysis shows that DLP was similarly composed of six monosaccharaides: mannose, ribose, glucuronic acid, glucose, xylose, and galactose. This result differed from that of CLP at molar ratios of 2.2, 1.2, 2.32, 2.0, 1.2, and 2.84, respectively.

### 3.4. FTIR Analysis

**Figure 3:**
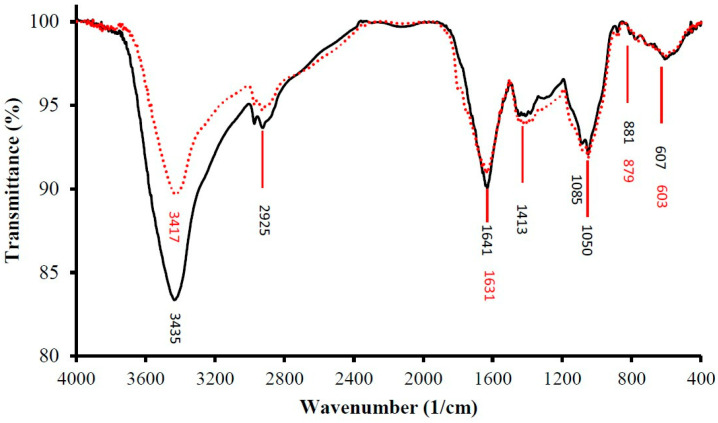
FTIR spectrum of CLP (solid black) and DLP (dotted red).

The infrared spectrogram (FTIR) of CLP, presented in Figure 3, revealed a typical absorption pattern of sugar, which further verified that CLP contained an abundant amount of polysaccharide. The absorption at 1050 cm^−1^ was attributed to the asymmetric C–O–C glycosides rings, indicating the presence of pyranose [7]. The peak at 1413 cm^−1^ was caused by COO– bonds, signifying the presence of uronic acids [33]. The peak at 2925 cm^−1^ indicates C–H stretching of –CH_2_–, and the peak at 1631 cm^−1^ was caused by C=O stretching of –NHCOCH_3_– in polysaccharides [34]. The major wide absorption peaks at 3417 cm^−1^ were caused by O–H and N–H bonds.

In comparison, the infrared spectrogram of DLP, as presented in Figure 3, showed a similar pattern as in CLP. The main peaks of DLP were almost the same as those of CLP, suggesting that no major functional group transformations were caused by degradation. The absorptions at 1085 and 1050 cm^−1^ were attributed to the asymmetric C–O–C glycosidic rings. The peaks at 1413 cm^−1^ were caused by COO– bonds. The peak at 2925 cm^−1^ indicates C―H stretching of –CH_2_–, and the peaks at 1641 cm^−1^ were caused by C=O stretching of –NHCOCH^3^– in polysaccharides. The major wide absorption peaks at 3435 cm^−1^ were caused by O–H and N–H bonds.

### 3.5. NMR Spectra Analysis

**Figure 4:**
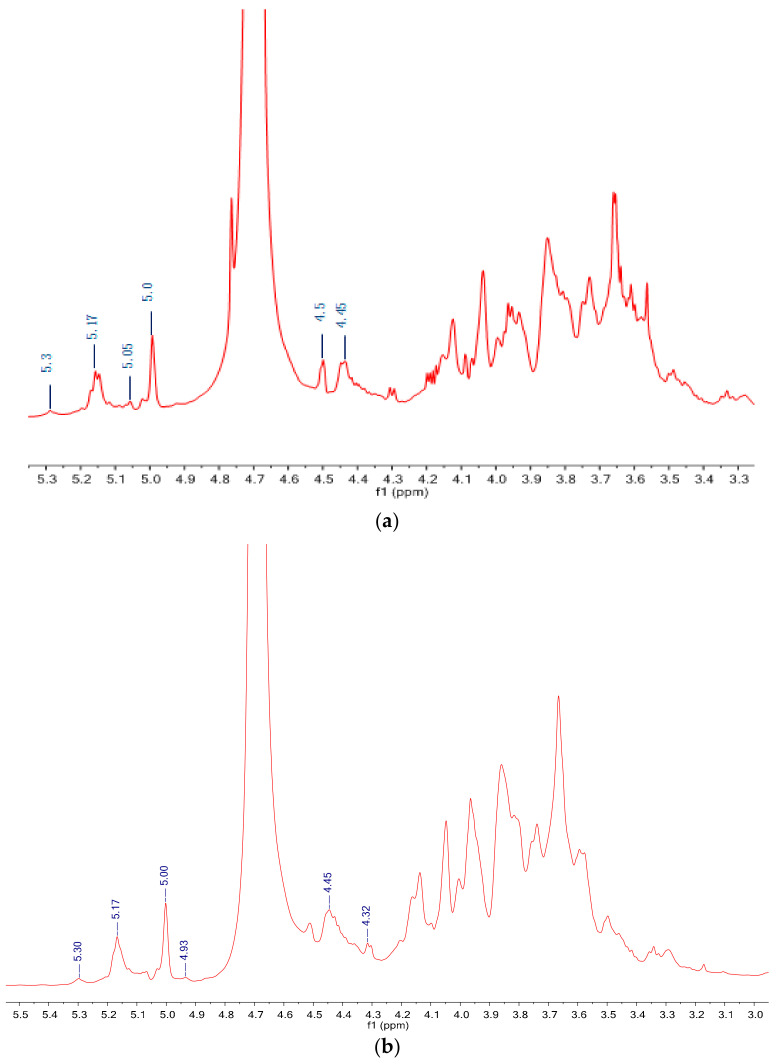
^1^H NMR spectra of (**a**) CLP and (**b**) DLP.

**Figure 5:**
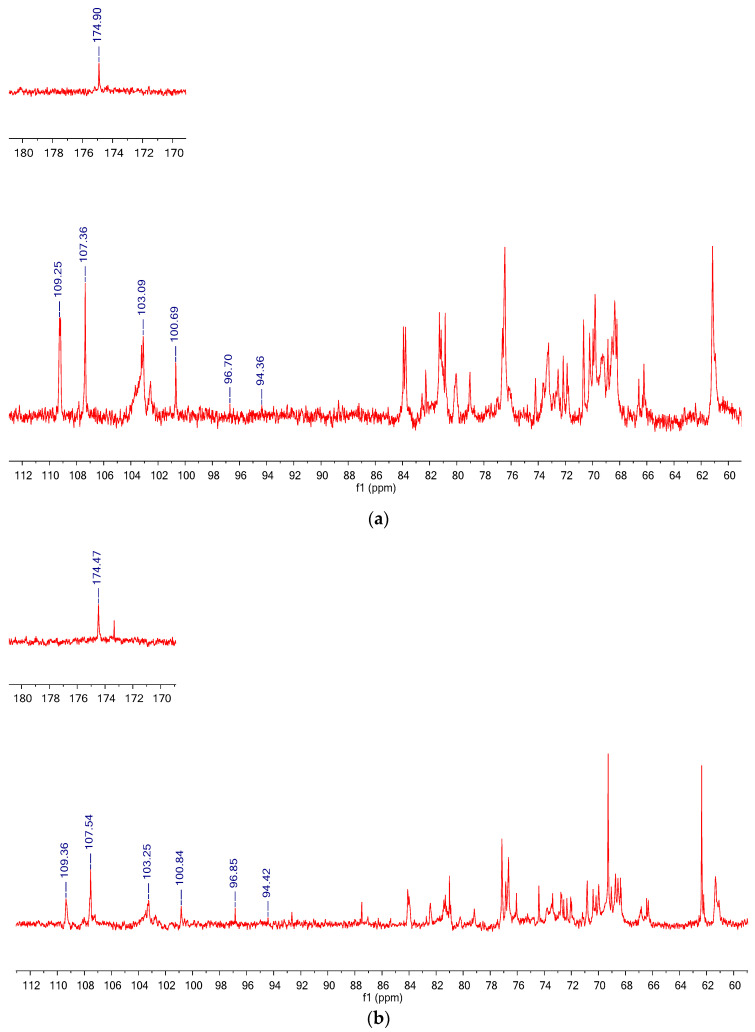
**^13^C** NMR spectra of (**a**) CLP and (**b**) DLP.

The ^1^H and ^13^C NMR spectra of CLP are represented in Figure 4a and Figure 5a, respectively. The ^1^H NMR spectrum of CLP showed six anomeric proton signals at δ 4.45, 4.5, 5.0, 5.05, 5.17, and 5.3 ppm. The anomeric proton signals from δ 4.45 to 4.5 ppm were attributed to the β-configurations, while δ 5.0 and 5.17 ppm were accredited to α-configurations.

^13^C NMR chemical shifts of CLP ranged between δ 60 and 110 ppm, Which represents a typical distribution of NMR signals in polysaccharides. CLP, shown in Figure 5a, has six major signals at δ 94.36, 96.70, 100.69, 103.09, 107.36, and 109.25 for six anomeric carbons.

^1^H and ^13^C spectra of DLP are shown in Figure 4b and Figure 5b, respectively. It could be seen that the spectra of DLP fairly resembled that of CLP. The anomeric proton signals of DLP were found at δ5.30, 5.17, 5.00, 4.93, 4.45, and 4.32; and the anomeric carbon signals of DLP were at δ109.36, 107.54, 103.25, 100.84, 96.85, and 94.42. Obviously, the signals of the anomeric region of DLP were almost identical to those of CLP, which demonstrated that CLP and DLP had similar glycosidic linkage patterns. Furthermore, the signals at δ174.47 showed that DLP contained uronic acid [35]. In summary, H_2_O_2_ and Vc degradation did not change the main structure of polysaccharides, and it was consistent with the GC and FTIR results above.

### 3.6. LP Molecular Weight and Conformation

**Figure 6:**
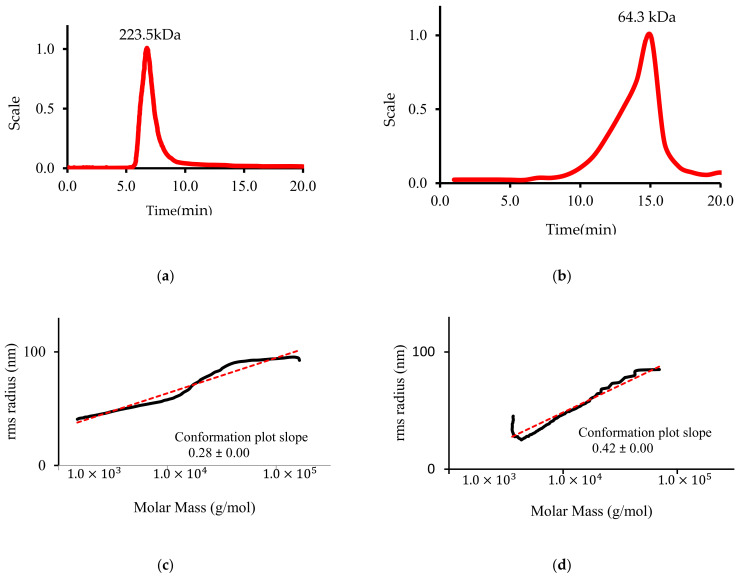
Molecular Mass and Radius of Gyration (Rg) where: (**a**) CLP Molecular Mass (**b**) DLP Molecular Mass (**c**) CLP Radius of Gyration (Rg) (**d**) DLP Radius of Gyration (Rg).

Mw and radius of gyration (Rg) of CLP were determined using HPGPC. Figure 6 below shows that CLP had a single symmetric peak and was a homogenous polysaccharide with Mw of 223.5 ± 1.13 kDa. This is more than that of 171.8 kDa, reported by [18], and lower than that of 250 kDa, reported by [22]. The Rg and the Rg vs. Mw curve slope of CLP was 42.4 ± 3.2 and 0.28 nm, respectively, suggesting that CLP has a compact globule conformation in a thermodynamically good solvent [18,22].

On the other hand, the Mw and radius of gyration (Rg) of DLP were determined to understand the degradation effect on LP chain conformation. The HPGPC result (Figure 6) showed that there was a significant decrease in Mw of DLP to 64.3 kDa, indicating that the molecular structure of CLP was severely affected by degradation using H_2_O_2_ and Vc. In addition, the Rg and the Rg vs. Mw curve slope of DLP increased to 51.3 ± 2.8 and 0.42 nm, respectively, indicating that DLP has a slack stretched irregular chain conformation [36].

In this study, CLP was further degraded using four different concentrations of H_2_O_2_ and Vc. Table 2 shows the Mw distribution determined for each concentration. It could be noticed that as the concentrations of H_2_O_2_ and Vc increased from 0 to 80 mM, the Mw decreased from 223.5 to 64.3 kDa. The degradation degree increased from 20% for DLP20 to 35%, 52.4%, and 71.2% for DLP40, DLP60, and DLP80, respectively. These results indicate that high H_2_O_2_ and Vc concentrations cause intensive destruction of polysaccharide chains.

### 3.7. LP Surface Morphology

**Figure 7:**
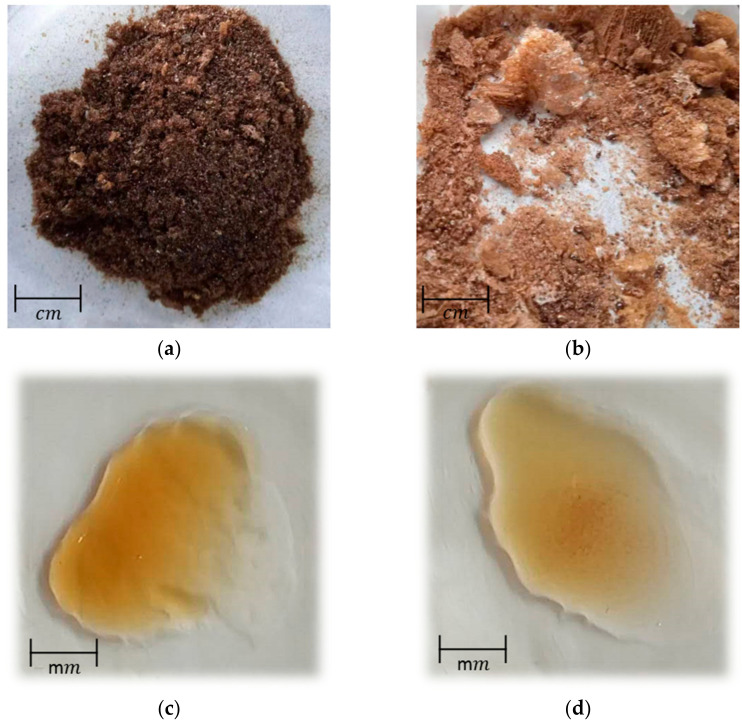

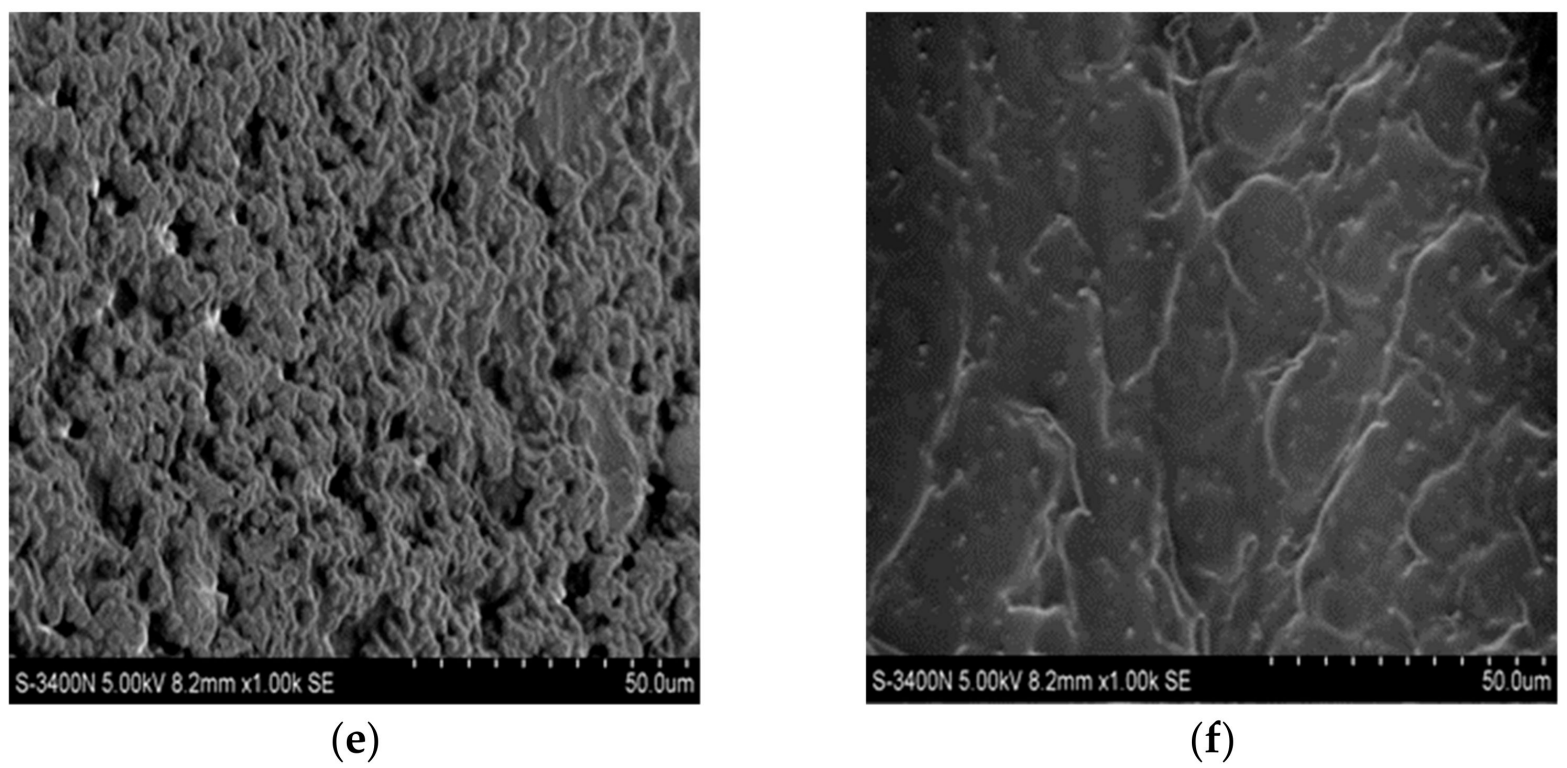
Images of (**a**) dry CLP (**b**) dry DLP (**c**) wet CLP (**d**) wet DLP (**e**) SEM image of CLP and (**f**) SEM image of DLP.

The surface morphology of CLP and DLP were considerably different, as shown by the SEM images results presented in Figure 7. CLP has a dark, viscous, thick, rough, porous appearance, with clearly visible lumps of aggregates including polysaccharide particles. In comparison, DLP has a lighter, spright, silky-smooth, and relatively uniform appearance. A previous study reported that the extraction, purification, and preparation conditions affect polysaccharide’s structure and surface morphology [37]. The changed surface morphology implied that the polysaccharide molecules had a significant transformation after degradation.

## 4. Discussion

Generally, Polysaccharide’s structure is the key for their various biological activities. Polysaccharide’s molecular weight, acid group content, monosaccharide composition, and main chain structure among other factors could greatly play an affective role in the biological activity of plant-derived polysaccharides [38,39,40,41]. In this study, CLP is the LL rich polysaccharide extract, which consisted of six monosaccharaides and was highly rich in minerals, with an abundant amount of uronic acid. DLP was obtained after CLP degradation using Vc and H_2_O_2_. Both LPs were highly purified and rich in soluble sugar, and degradation had no significant effect on protein, total sugar, and uronic acid contents. Moreover, degradation had no major effect on LP monosaccharaides composition. Gas chromatograph spectroscopic analysis showed that both LPs were composed of six monosaccharaides: mannose, ribose, glucuronic acid, glucose, xylose, and galactose. Differences in LP monosaccharide components reported by [12,18,22] could be attributed to processing methods, separation procedures, and purification conditions [42]. In addition, FTIR and NMR results had shown that the main chain structures of CLP and DLP remain intact, but nonetheless, the absorption peak intensities of the characteristic functional group of the polysaccharides before and after degradation were different. This is mainly caused by the hydroxyl radicals produced by H_2_O_2_ degradation reaction, which can react with the polysaccharide glycosidic bonds and cut them, resulting in the change of side chain structure but not the main chain structure in CLP. In summary, degradation only leads to changes of side groups, and no distinct change takes place in the structures of the main chain.

CLP has a high molecular weight and large molecular volume, which affect its biological activity. This is closely related to the hydroxyl group formation and the chain single-helix structure. Moreover, CLP has stronger inter-molecular hydrogen bonds that form a high compact structure, which in turn result in less exposure of effective active groups [43]. Reports also indicate that the polysaccharide chain has a reduction and nonreducing ends. Lesser ends were found in the higher molecular weight polysaccharides, hindering biological activity [44]. This was reported by Zhang et. al., where *Lycium barbarum* L. polysaccharides with low molecular weight (10.2 kDa) have anticancer activity, whereas those with high molecular weight (6.50 × 10^3^ kDa) have no anticancer activity [45].

Degradation of polysaccharide, as in the case of DLP, could significantly alter its conformation and can thus effectively improve its biological activities. DLP with a lower molecular weight has a smaller branching degree, providing sufficient spatial extent to form a regular helical structure, which favours the exposure of more hydroxyl groups to the surface of the conformation and is good for exerting bioactivity functions. However, this effect could cut both ways, as excessive degradation, which could lead to much lower molecular weights, could also negatively impact polysaccharides bioactivities. In significantly lower molecular weight polysaccharide, the unique bond linking method and conformation based on intramolecular hydrogen bond will be destroyed. The carbonyl group in low molecular weight polysaccharide would change from a ring formation to be in an open chain. The destruction of the ring formation will break the hydrogen bond structure of the polysaccharide molecule, leading to reduced bioactivity. This was reported by [46], as high molecular weight polysaccharides from Opuntia have shown a better immunoregulatory effect than that of Opuntia polysaccharides with a low molecular weight.

## 5. Conclusions

This study demonstrated a comprehensive analysis on the degradation effect on *Lycium barbarum* L. leaves’ polysaccharide. Both crude and degraded polysaccharides were further characterized and the proximate composition, monosaccharides components, molecular weight, structural configuration, and surface morphology were analysed and compared. Degradation had no major effect on LP proximate composition and monosaccharides components. Analysis showed that both LPs were composed of the same monosaccharaides. Furthermore, no major functional group transformations were caused by degradation; degradation only leads to changes in the side groups, with no distinct changes occurring in the structures of the main chain. However, the chemical degradation of polysaccharide using H_2_O_2_ and Vc decreased the Mw of polysaccharide by 70%. The mechanism underlying this degradation process comprises the breakdown of glycosidic links caused by hydroxyl radicals originating from the reaction system of H_2_O_2_ and Vc, which react with hydrogen atoms of polysaccharides. Hence, compared with other degradation methods, this chemical degradation by Vc and H_2_O_2_ is easily controlled, more efficient, has highly moderate reaction conditions, and causes mildly negligible damage to sugar structures. This study will promote further research on LP structure–activity relationships, which could be utilized as an excellent source of polysaccharides.

## Figures and Tables

**Table 1:** Proximate compositions and mineral content of CLP and DLP.

Content	CLP	DLP
Mw (kDa)	223.5 ± 1.13	64.3 ± 2.03
Uronic Acid (%)	13.7 ± 1.7	15.2 ± 0.7
Protein (%)	2.5 ± 0.1	2.67 ± 0.36
Total Sugar (%)	94.0 ± 7.3	89.2 ± 7.9
Mg (mg/g)	3.96 ± 0.22 a	3.63 ± 0.40 a
Fe (mg/g)	2.29 ± 0.32 b	1.96 ± 0.17 a
Zn (mg/g)	1.51 ± 0.16 a	1.42 ± 0.35 b
Ca (mg/g)	8.80 ± 1.1 a	7.5 ± 0.2 b

Values are means ± SD (n = 3). Values in the same row represented by different letters differ significantly (*p* < 0.05).

**Table 2:** Mw under different concentrations of H_2_O_2_ and Vc.

	H_2_O_2_ (mM)	Vc (mM)	Mw (kDa)
CLP	-	-	223.5 ± 1.13 a
DLP	20	20	187.7 ± 0.53 a
	40	40	144.9 ± 2.40 b
	60	60	96.26 ± 1.71 c
	80	80	64.3 ± 2.03 d

Values are means ± SD (n = 3). Values in the same row represented by different letters differ significantly (*p* < 0.05).

## Data Availability

The data presented in this study are available on request from the corresponding author.
